# 

**Published:** 1880-04

**Authors:** 


### CONTENTS:

ARTICLE I.—New Methods of Mounting Artificial Teeth.......... 529
ARTICLE II.—Sale of Diplomas.......... 539
ARTICLE III.—Lectures on Operative Dental Surgery and Therapeutics. By W. Finley Thompson, M. D., D. D. S.......... 544
ARTICLE IV.—Mechanical Dentistry. By Edgar Swain, D. D S.......... 555
ARTICLE V.—Tooth-Caries of Pregnancy—Its Cause and Treatment. By Ed. C. Kirk, D. D. S.......... 560
ARTICLE VI.—Poisons and their Antidotes. By Dr. Th. Schlosser.......... 565
Dental Department of Vanderbilt University.......... 568
Georgia State Dental Society.......... 568

### EDITORIAL, ETC.

Law Regulating the Practice of Dentistry in Georgia.......... 569

### BIBLIOGRAPHICAL.

A Treatise on Oral Deformities as a Branch of Mechanical Surgery.......... 571
Headaches; Their Nature, Cause and Treatment.......... 572
Brain Work and Over-work.......... 572
Sore Throat; Its Nature, Varieties and Treatment.......... 573
Petermann's Dental Almanac for 1880.......... 573
Our Homes.......... 573

### MONTHLY SUMMARY.

Chloral as an Anæsthetic for Children.......... 574
Death from Chloroform in a Dentist's Chair.......... 574
The Effects of Tobacco.......... 575
Effects of Tea on the System.......... 576

